# Context matters – Daxx and Atrx are not robust tumor suppressors in the murine endocrine pancreas

**DOI:** 10.1242/dmm.049552

**Published:** 2022-08-26

**Authors:** Chang Sun, Jeannelyn S. Estrella, Elizabeth M. Whitley, Gilda P. Chau, Guillermina Lozano, Amanda R. Wasylishen

**Affiliations:** 1 Department of Genetics, The University of Texas MD Anderson Cancer Center, Houston, TX, 77030 USA; 2 Genetics and Epigenetics Program, The University of Texas MD Anderson Cancer Center UTHealth Graduate School of Biomedical Sciences, Houston, TX 77030, USA; 3 Department of Anatomic Pathology, The University of Texas MD Anderson Cancer Center, Houston, TX 77030, USA; 4 Department of Veterinary Medicine and Surgery, The University of Texas MD Anderson Cancer Center, Houston, TX 77030, USA

**Keywords:** Daxx, Atrx, Men1, Pancreatic neuroendocrine tumor, Mouse model

## Abstract

Genome sequencing has revealed the importance of epigenetic regulators in tumorigenesis. The genes encoding the chromatin remodeling complex DAXX:ATRX are frequently mutated in pancreatic neuroendocrine tumors; however, the underlying mechanisms of how mutations contribute to tumorigenesis are only partially understood, in part because of the lack of relevant preclinical models. Here, we used genetically engineered mouse models combined with environmental stress to evaluate the tumor suppressor functions of Daxx and Atrx in the mouse pancreas. *Daxx* or *Atrx* loss, alone or in combination with *Men1* loss, did not drive or accelerate pancreatic neuroendocrine tumorigenesis. Moreover, *Daxx* loss did not cooperate with environmental stresses (ionizing radiation or pancreatitis) or with the loss of other tumor suppressors (*Pten* or *p53*) to promote pancreatic neuroendocrine tumorigenesis. However, owing to promiscuity of the Cre promoter used, hepatocellular carcinomas and osteosarcomas were observed in some instances. Overall, our findings suggest that Daxx and Atrx are not robust tumor suppressors in the endocrine pancreas of mice and indicate that the context of a human genome is essential for tumorigenesis.

This article has an associated First Person interview with the first author of the paper.

## Abstract (precis)

**Summary:** Daxx and Atrx are not robust tumor suppressors in murine endocrine pancreas, suggesting that the impact of human-specific features (telomere length, transposable elements) on tumorigenesis is not easily recapitulated in mice.

## INTRODUCTION

Recent genome sequencing efforts have revealed candidate drivers of tumorigenesis and, in some cases, implicated novel genes as tumor suppressors. The exome sequencing of ten sporadic pancreatic neuroendocrine tumors (PanNETs) identified recurrent and mutually exclusive loss-of-function mutations in *DAXX* and *ATRX* for the first time (Jiao et al., 2011). Sanger sequencing of a validation set of 58 additional PanNETs demonstrated mutation frequencies of 25% and 18%, respectively. A subsequent whole-genome sequencing study confirmed these results with *DAXX* mutations in 22% (22 of 98), and *ATRX* mutations in 11% (11 of 98), of tumors (Scarpa et al., 2017).

The mutually exclusive pattern of *DAXX* and *ATRX* loss suggests that these mutations impinge on the same molecular pathway. Together, DAXX and ATRX form a chaperone complex for the histone 3.3 variant and mediate the deposition of H3.3 at heterochromatic regions of the genome, including telomeres, pericentromeric regions and endogenous retroviral loci (Elsasser et al., 2012; Elsasser et al., 2015; He et al., 2015; Lewis et al., 2010; Drane et al., 2010; Sadic et al., 2015). Remarkably, there is a near-perfect association between PanNETs with *DAXX* or *ATRX* mutations and tumors that activate the telomerase-independent alternative lengthening of telomeres (ALT) pathway (Heaphy et al., 2011), indicating that telomere dysfunction may contribute to tumorigenesis. *ATRX* loss, however, is insufficient to induce ALT *in vitro* (Brosnan-Cashman et al., 2018), while its overexpression can reverse ALT phenotypes in U2OS osteosarcoma cells (Clynes et al., 2015).

Understanding the molecular consequences downstream of *DAXX* or *ATRX* loss requires relevant preclinical experimental models. To date, cell line models of PanNETs have been limited and remain controversial. The established lines may not express neuroendocrine markers and contain mutations that, although common in other cancers including pancreatic ductal adenocarcinomas, are not frequent in PanNETs (Hofving et al., 2018; Vandamme et al., 2015). This includes mutations in the RAS family oncogenes and *TP53*. Genetically engineered mouse models (GEMMs) provide a powerful platform to study gene function *in vivo*. Germline knockout models of both *Daxx* and *Atrx* are early embryo lethal, but with notably different phenotypes (Michaelson et al., 1999; Garrick et al., 2006). *Daxx*-null mice die between embryonic day (E)6.5 and E8.5 and exhibit apoptosis. This increased apoptosis is also observed in isolated embryonic stem cells (Michaelson et al., 1999). *Atrx*-null mice die at a similar time point and show no robust induction of apoptosis, but rather exhibit defects in trophoblast development (Garrick et al., 2006). Combined, these results suggest that independent functions of both proteins are important during murine embryonic development. This early embryonic lethality, however, necessitated the development of conditional alleles to allow for temporal and spatial control of gene loss. Conditional *Atrx* knockout mice (*Atrx^fl^*), with exon 18 flanked by loxP sites, have been used extensively to model ATRX syndrome, with tissue-specific knockout in the organs in which this rare germline disorder manifests, including neurons and limb mesenchyme (Berube et al., 2005; Solomon et al., 2013). We recently developed a conditional *Daxx* allele (*Daxx^fl^*), which revealed an important role for Daxx in regulating exocrine tissue homeostasis, chromatin accessibility and silencing endogenous retroviral loci *in vivo* (Wasylishen et al., 2020). Notably, this work did not reveal robust phenotypic changes in the endocrine pancreas as a consequence of *Daxx* loss.

Here, we used these conditional alleles to specifically and comprehensively evaluate Atrx and Daxx as endocrine tumor suppressors in the mouse pancreas. Remarkably, using a combination of different Cre driver lines, environmental stressors and cooperating genetic lesions, we found no evidence that Atrx or Daxx function as tumor suppressors in the endocrine pancreas of mice. These results strongly indicate that a human genome is essential to promote tumorigenesis downstream of *ATRX* or *DAXX* loss.

## RESULTS

### *Daxx* or *Atrx* loss from β cells is insufficient to drive pancreatic neuroendocrine tumorigenesis

**Fig. 1.**
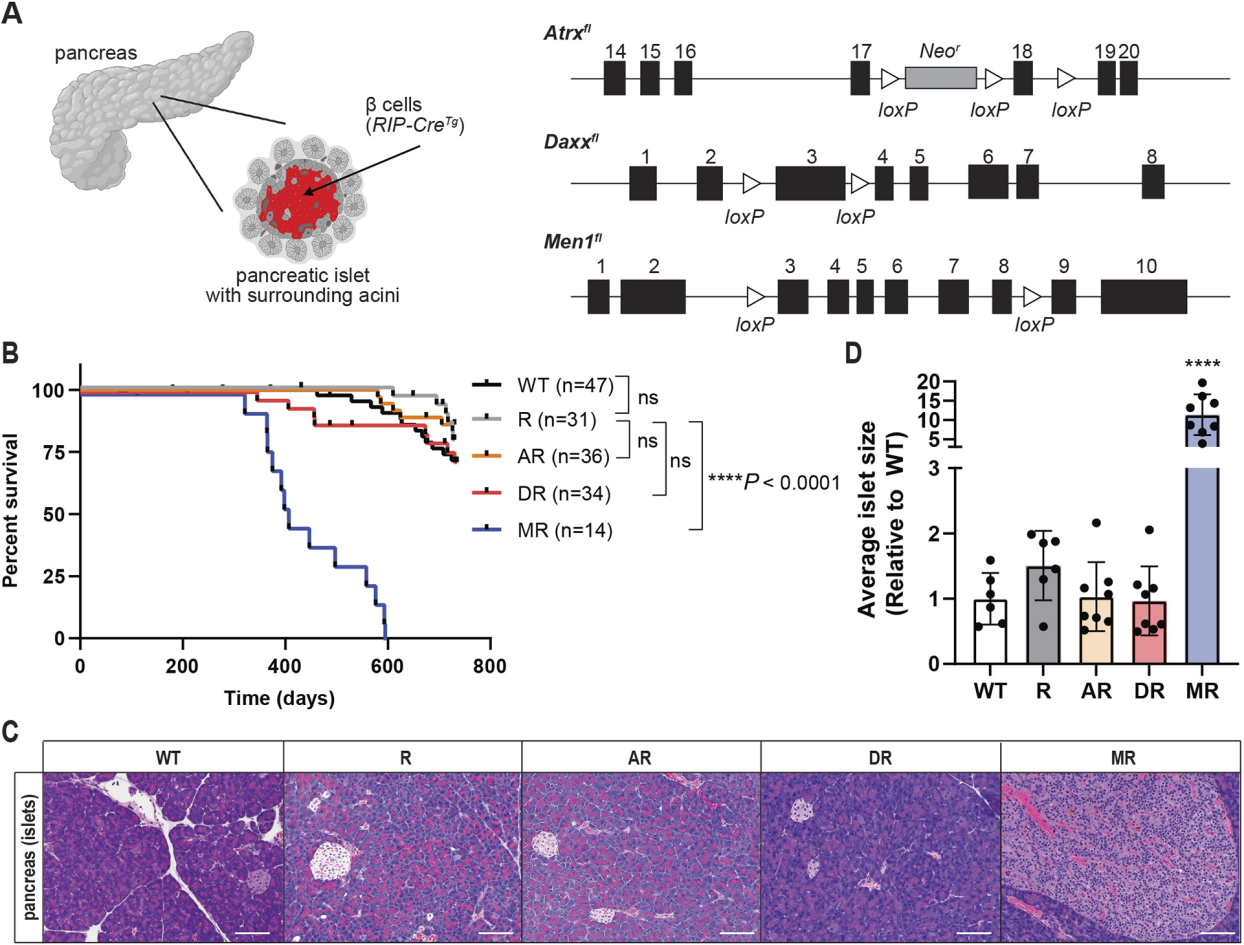
***Daxx* or *Atrx* loss from β cells is insufficient to drive pancreatic neuroendocrine tumorigenesis in mice.** (A) Schematic representation (created with biorender.com) of the pancreas, with β-cell targeting achieved by the *RIP-Cre^Tg^* (R), and *Atrx* (A), *Daxx* (D) and *Men1* (M) conditional alleles. (B) Kaplan–Meier survival analysis. ns, not significant, ****P<0.0001, log-rank (Mantel–Cox) test. WT, wild-type; R, *RIP-Cre^Tg^*; AR, *Atrx^fl/fl^RIP-Cre^Tg^* or *Atrx^fl/Y^RIP-Cre^Tg^*; DR, *Daxx^fl/fl^RIP-Cre^Tg^*; MR, *Men1^fl/fl^RIP-Cre^Tg^.* (C) Representative Hematoxylin and Eosin (H&E) sections of pancreatic islets from mice of the different genotypes. Images taken at 10× magnification, scale bars: 100 µm. (D) Average islet size per mouse presented relative to the average islet size of WT mice. ****P<0.001, one-way ANOVA with Dunnett's multiple comparisons test compared with WT.

One of the first transgenic mouse models of cancer developed was a model of PanNETs, with the rat insulin promoter (RIP) driving transgenic expression of the SV40 large T antigen in β cells (Hanahan, 1985). RIP-Tag mice rapidly develop highly penetrant functional PanNETs (which are predominantly insulinomas), and mice succumb to hypoglycemia induced by the high levels of insulin produced by the tumors. RIP has also been used to drive expression of Cre recombinase to delete *Men1* specifically from β cells (Crabtree et al., 2003). The resulting mice also develop highly penetrant insulinomas, but at much longer latency. To evaluate potential tumor suppressor function(s) of Atrx or Daxx in murine β cells, we crossed conditional alleles to the *RIP-Cre^Tg^* line and established a cohort of *Atrx^fl/fl^RIP-Cre^Tg^* or *Atrx^fl^/Y RIP-Cre^Tg^*, as *Atrx* is on the X chromosome (abbreviated AR), and *Daxx^fl/fl^RIP-Cre^Tg^* (DR) mice compared with both *RIP-Cre^Tg^* (R) and Cre negative [wild-type (WT)] controls (Fig. 1A). We also included *Men1^fl/fl^RIP-Cre^Tg^* (MR) mice as a positive control for pancreatic neuroendocrine tumorigenesis. The established cohorts were aged, and when evaluated to a 2-year end point, neither *Atrx* nor *Daxx* loss significantly reduced survival compared with Cre-expressing controls (Fig. 1B). Additionally, the two control genotypes (R and WT) were indistinguishable from each other. As expected, MR mice had a significantly reduced survival (*P*<0.0001, Fig. 1B) and significantly reduced blood glucose at necropsy, indicating the presence of insulinomas (Fig. S1A). The blood glucose measurements of R mice were normal (mean, 101 mg/dl), indicating no significant effects of the *RIP-Cre^Tg^* transgene on glucose homeostasis in this cohort. We next conducted histological analysis of pancreas sections and found no evidence of early lesions in either AR or DR mice (Fig. 1C). Finally, quantification of islet size revealed no significant change in average islet size compared with that of controls (Fig. 1D). MR mice, on the other hand, displayed extensive islet hyperplasia and developed well-differentiated PanNETs composed of a proliferation of small- to medium-sized cells with small, uniform, round to oval nuclei, with coarsely clumped chromatin and eosinophilic, finely granular cytoplasm, forming large, coalescing nests (10× compared to WT control, on average) (Fig. 1C,D). As previously reported, MR mice also developed pituitary tumors, morphologically similar to the PanNETs (Fig. S1B) (Crabtree et al., 2003). Additionally, combined loss of *Daxx* and *Men1* in β cells driven by *RIP-Cre^Tg^* (DMR) was indistinguishable from *Men1* loss alone (MR; Fig. S1C-E).

### *Atrx* loss does not accelerate *Men1* loss-driven PanNETs

Emerging data indicate that *DAXX* and *ATRX* mutant PanNETs exhibit expression and epigenetic signatures of α cells (Cejas et al., 2019; Chan et al., 2018). To investigate *Daxx* loss across all epithelial cells of the pancreas, our previous work combined *Daxx* and *Men1* loss driven by the *Pdx1-Cre^Tg^* (P) driver. Although we observed homeostatic defects in the exocrine pancreas, we identified no significant changes in survival or endocrine phenotypes when we compared loss of *Daxx* and *Men1* to *Men1* loss alone (Wasylishen et al., 2020). However, although *Daxx* loss does not cooperate with *Men1* loss to drive PanNETs in mice driven by either *RIP-Cre^Tg^* (Fig. S1) or *Pdx1-Cre^Tg^* (Wasylishen et al., 2020), germline deletions of *Atrx* and *Daxx* do not phenocopy each other (Michaelson et al., 1999; Garrick et al., 2006), indicating that *Atrx* loss should also be evaluated in the sensitized context of *Men1* deficiency.

**Fig. 2.**
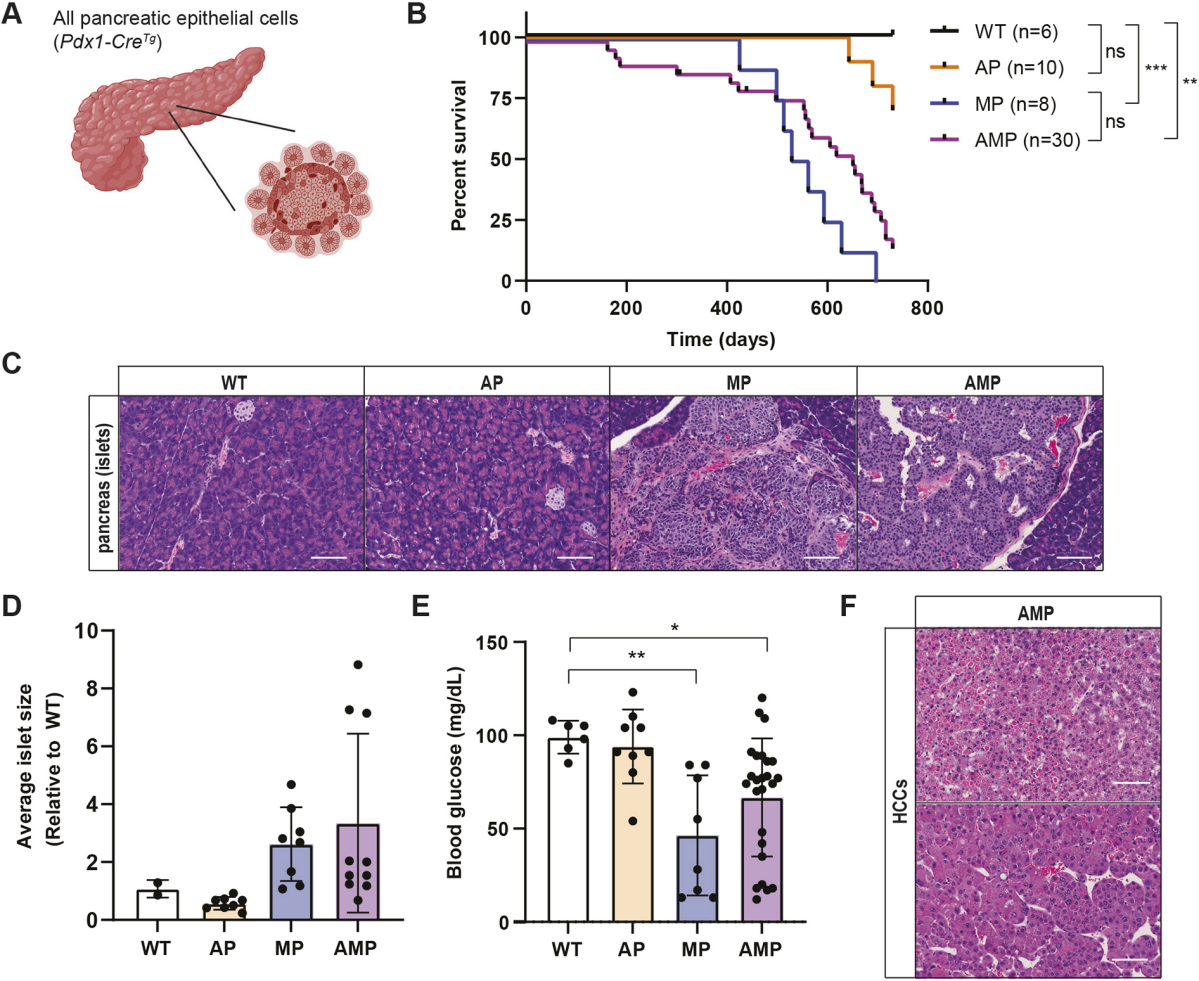
***Atrx* loss does not accelerate *Men1* loss-driven pancreatic neuroendocrine tumors (PanNETs) in mice.** (A) Schematic representation (created with biorender.com) of the pancreas, with all epithelial cells targeted by *Pdx1-Cre^Tg^* (P). (B) Kaplan–Meier survival analysis. ns, not significant; **P<0.01, ***P<0.001, log-rank (Mantel–Cox) test. WT, wild-type; AP, *Atrx^fl/fl^Pdx1-Cre^Tg^* or *Atrx^fl/Y^Pdx1-Cre^Tg^*; MP, *Men1^fl/fl^Pdx1-Cre^Tg^*; AMP, *Atrx^fl/fl^Men1^fl/fl^Pdx1-Cre^Tg^* or *Atrx^fl/Y^Men1^fl/fl^Pdx1-Cre^Tg^*. (C) Representative H&E sections of pancreatic islets from mice of the different genotypes. Images taken at 10× magnification, scale bars: 100 µm. (D) Average islet size per mouse presented relative to the average islet size of WT mice from Fig. 1D. (E) Non-fasting blood glucose measurements from mice at necropsy with survival >200 days. *P<0.05, **P<0.01, one-way ANOVA with Dunnett's multiple comparisons test compared with WT. (F) Representative H&E sections of hepatocellular carcinomas (HCCs) identified in AMP mice. Images taken at 10× magnification, scale bars: 100 µm.

We then evaluated cooperation between *Atrx* and *Men1* loss throughout all epithelial cells of the pancreas, driven by *Pdx1-Cre^Tg^* (AMP) compared with loss of either gene alone (AP and MP) and Cre-negative littermate controls (Fig. 2A). Our previous work included *Pdx1-Cre^Tg^* controls (Wasylishen et al., 2020), which were aged in the facility during the same time period and had normal survival, normal non-fasting blood glucose at necropsy (mean, 97 mg/dl) and average islet size compared with Cre-negative control mice. Loss of *Atrx* was well tolerated in the pancreas and had no effect on overall survival (Fig. 2B). Additionally, the survival of AMP mice was indistinguishable from that of MP mice, with similar morbidity associated with hypoglycemia and functional PanNETs (Fig. 2C-E). To confirm that *Pdx1-Cre^Tg^* targeted α cells, we used a Cre reporter allele (*Rosa26^LSL−tdTomato/+^*) that is only expressed upon Cre recombination. We observed co-expression of tdTomato and glucagon in islet cells from *Pdx1-Cre^Tg^ Rosa26^LSL−tdTomato^* mice (Fig. S2A). As an additional control, we also used the *Pdx1-Cre^Tg^* driver line to validate the conditional loss of expression of *Daxx*, *Atrx* and *Men1* in DP, AP and MP pancreases. Western blot analyses of total pancreas lysates revealed an average of 65-70% decreases in protein expression across the panel (Fig. S2B-D). Similar to *Daxx* cohorts (Wasylishen et al., 2020) (Fig. S1C-E), *Atrx* loss alone or in the background of *Men1* deficiency did not promote pancreatic neuroendocrine tumorigenesis (Fig. 2C,D).

Although *Atrx* loss did not cause or accelerate PanNETs in the sensitized background of homozygous *Men1* loss, we did observe similar exocrine phenotypes as with *Daxx* loss (Wasylishen et al., 2020). Metaplastic areas were present in more AMP mice (7/11; 63.6%) than in MP mice (3/8; 37.5%). Cystic lesions were also present in 5/11 (45.5%) of evaluated AMP mice and resembled the lesions identified in our previous Daxx studies (Wasylishen et al., 2020). This result is also consistent with independent work demonstrating that *Atrx* loss from acinar cells leads to persistent damage and metaplasia following caerulein-induced pancreatitis (Young et al., 2019). There were additionally two unexpected outcomes in mice with the combined loss of *Atrx* and *Men1* driven by *Pdx1-Cre^Tg^*. First, two of the mice (2/30 6.7%) became moribund at ∼6 months of age (178 and 187 days), presenting with lethargy, weight loss and hunching. Notably, these mice had extremely elevated non-fasting blood glucose (>600 mg/dl and 492 mg/dl), indicative of diabetes. These two blood glucose measurements were excluded from the data presented in Fig. 2E. Second, 7/30 (23%) of necropsied AMP mice had macroscopic liver lesions on necropsy. Six of these lesions underwent pathological evaluation and the majority (5/6) were identified as hepatocellular carcinoma (HCC), showing abnormal proliferation of hepatocytes with nuclear enlargement, intranuclear inclusions, prominent nucleoli and pleomorphism. Some neoplastic hepatocytes exhibited features mimicking disease states, including eosinophilic intracytoplasmic globules mimicking alpha-1 antitrypsin deficiency (Fig. 2F, top) and steatohepatitis. In these areas, the cells were associated with thickened trabeculae (Fig. 2F, bottom) and unpaired arteries, and lacked portal tracts. The remaining lesion was a lymphoma. Although lymphomas are relatively common in aged mice, we did not identify HCCs in AP mice or any of our previous *Men1* or *Daxx* cohorts. Notably, the *Pdx1-Cre^Tg^* line has reported activity in other cells types, including the bile ducts (Magnuson and Osipovich, 2013). Collectively, these two observations suggest the potential for genetic interaction between *Men1* and *Atrx*, and in tissues exclusive of the pancreas.

### *Daxx* loss does not cooperate with environmental stresses to induce pancreatic neuroendocrine tumorigenesis

**Fig. 3.**
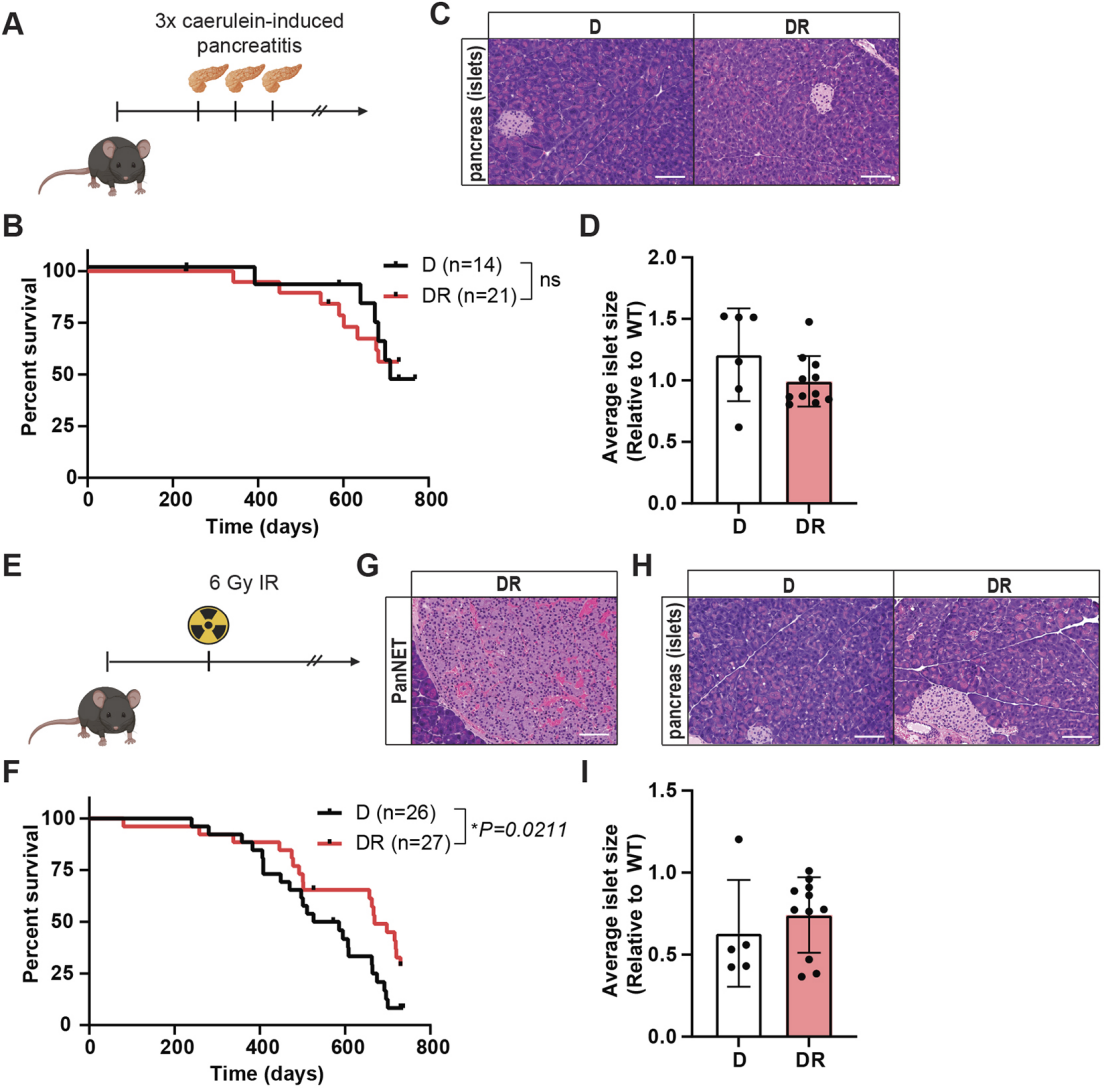
***Daxx* loss does not cooperate with environmental stresses to induce pancreatic neuroendocrine tumorigenesis.** (A) Schematic representation (created with biorender.com) of caerulein-induced pancreatitis induction. (B) Kaplan–Meier survival analysis. ns, not significant, log-rank (Mantel–Cox) test. D, *Daxx^fl/fl^*; DR, *Daxx^fl/fl^RIP-Cre^Tg^*. (C) Representative H&E sections of pancreatic islets from mice of the different genotypes. Images taken at 10× magnification, scale bars: 100 µm. (D) Average islet size per mouse presented relative to the average islet size of WT mice from Fig. 1D. (E) Schematic representation (created with biorender.com) of radiation treatment. (F) Kaplan–Meier survival analysis. *P<0.05, log-rank (Mantel–Cox) test. (G) H&E section of a microscopic pancreatic neuroendocrine tumor PanNET. Image taken at 10× magnification, scale bar: 100 µm (H) Representative H&E sections of pancreatic islets from mice of the different genotypes. Images taken at 10× magnification, scale bars: 100 µm. (I) Average islet size per mouse presented relative to the average islet size of WT mice from Fig. 1D.

Single genetic changes are often insufficient to promote tumorigenesis and require cooperating environmental stress or additional genetic changes. For example, activating mutations in *KRAS* are the most frequent alterations in pancreatic ductal adenocarcinomas (PDAC), yet inducing a *Kras* mutation in the epithelial cells of the pancreas of mice is insufficient to drive robust tumorigenesis. When a *Kras* mutation is combined with experimentally induced inflammation, mice rapidly develop precancerous pancreatic intraepithelial neoplasia (PanIN) lesions and PDAC (Guerra et al., 2007). Although there is no reported clinical association between pancreatitis and PanNETs, there are associations between chronic inflammation and PDAC (Hausmann et al., 2014; Alhobayb et al., 2021). Moreover, the microenvironment can strongly influence tumorigenesis. Mouse models with genetic induction of chronic inflammation in the context of *p53* (also known as *Trp53*) deficiency develop pancreas tumors, and the tumors that arise are of mixed cell types, including a neuroendocrine compartment, based on immunohistochemical staining for chromogranin A and synaptophysin (Swidnicka-Siergiejko et al., 2017). To evaluate whether tissue stress through chronic pancreatitis could cooperate with *Daxx* loss to promote tumorigenesis in β cells, we treated DR and Cre-negative littermate control (D) mice with the cholecystokinin analog caerulein. These treatments were six intraperitoneal injections administered hourly on each of 2 consecutive days (12 total), repeated three times with a 2-month interval between treatments to mimic chronic pancreatitis (Fig. 3A). We confirmed the induction of pancreatitis in independent mice by histological analysis 48 h following one round of caerulein injections. Caerulein-treated mice exhibited the expected immune cell infiltration and acinar-to-ductal metaplasia, which were not present in the PBS-treated controls (Fig. S3). As expected, based on the regenerative response of the pancreas and that *Daxx* loss was restricted to β cells, pancreatitis and the associated damage was completely resolved by the end point of the study, and we observed no evidence of persistent metaplasia. Additionally, DR and D mice exhibited no significant differences in survival or islet phenotypes (Fig. 3B-D).

We previously used a germline *Daxx*-null allele (*Daxx^Δ3^*) to evaluate the consequences of *Daxx* heterozygosity *in vivo* (Wasylishen et al., 2018). We treated a cohort of *Daxx^Δ3^*^/+^ mice with a low-dose ionizing radiation (IR) as an environmental stress known to induce mutations and promote tumorigenesis. One mouse in this cohort developed a small PanNET. We therefore evaluated whether homozygous loss of *Daxx* from β cells (DR) might cooperate with IR to drive tumorigenesis. A cohort of DR and D mice was established and treated with one sublethal dose of 6 Gy IR (Fig. 3E). Although IR treatment generally did decrease the overall survival of mice compared with untreated mice, DR mice had a slight increase in overall survival compared with that of D mice (*P*=0.0211) (Fig. 3F), but no notable changes in the frequencies or distributions of extra-pancreatic pathologies or other tumors. Similar to the pancreatitis cohort, analysis of pancreas sections identified only a single microscopic PanNET (Fig. 3G), and, overall, there was no significant change in average islet size (Fig. 3H,I), indicating no robust cooperation between *Daxx* loss and IR in the endocrine pancreas of mice.

### *Daxx* loss does not cooperate with the loss of other established tumor suppressors to promote PanNET development

**Fig. 4.**
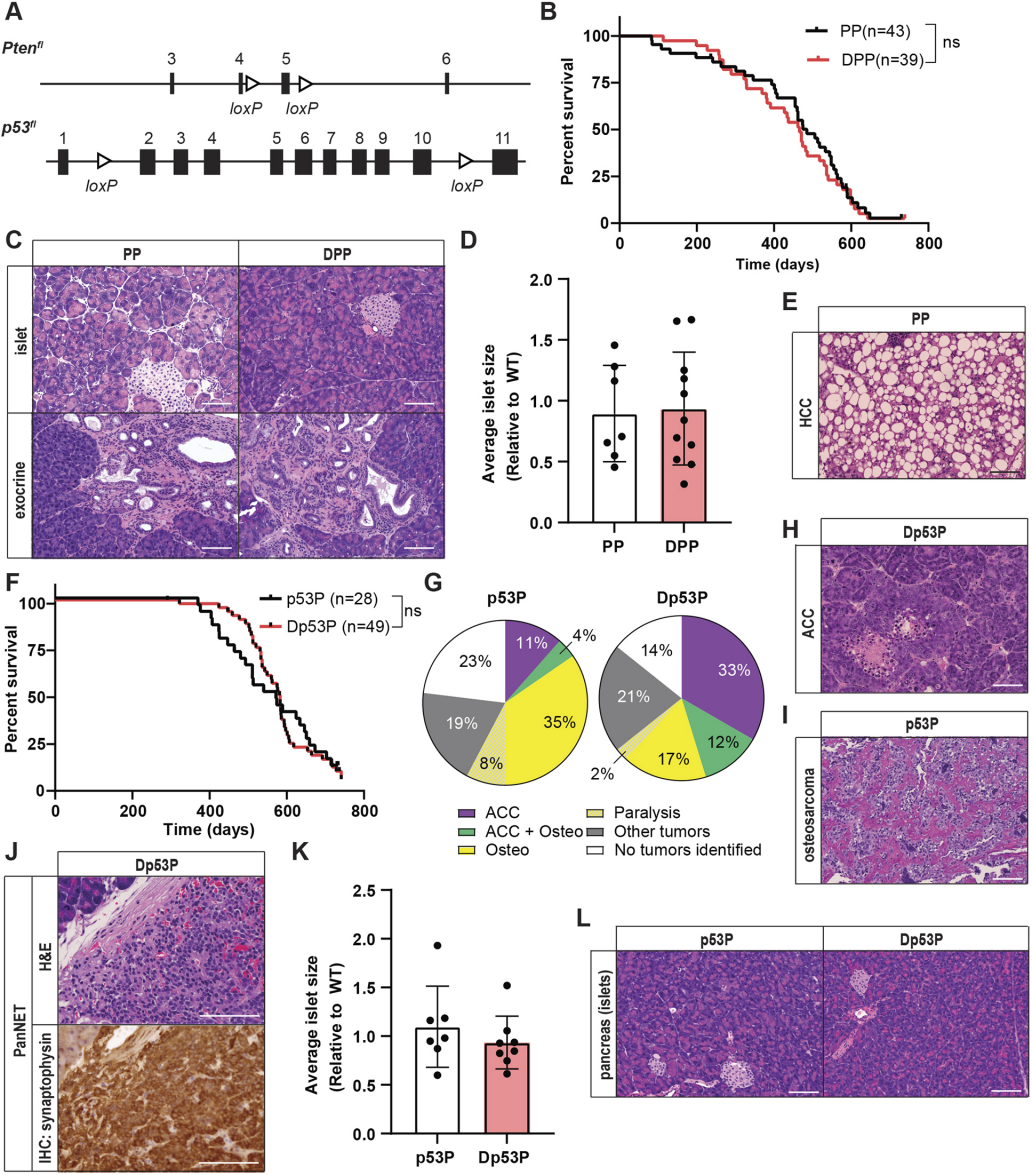
***Daxx* loss does not cooperate with the loss of other established tumor suppressors to promote PanNET development.** (A) Schematic representation of conditional *Pten* (P) and *Trp53* (p53) alleles. (B) Kaplan–Meier survival analysis. ns, not significant, log-rank (Mantel–Cox) test. PP, *Pten^fl/fl^Pdx1-Cre^Tg^*; DPP, *Daxx^fl/fl^Pten^fl/fl^Pdx1-Cre^Tg^*. (C) Representative H&E sections of pancreatic islets (top row) and pancreatic exocrine tissue (bottom row) from mice of the different genotypes. Images taken at 10× magnification, scale bars: 100 µm. (D) Average islet size per mouse presented relative to the average islet size of WT mice from Fig. 1D. (E) Representative H&E section of HCC mimicking steatohepatitis from one PP mouse. Image taken at 10× magnification, scale bar: 100 µm. (F) Kaplan–Meier survival analysis. ns, not significant, log-rank (Mantel–Cox) test. p53P, *p53^fl/fl^Pdx1-Cre^Tg^*; Dp53P, *Daxx^fl/fl^p53^fl/fl^Pdx1-Cre^Tg^*. (G) Pie charts representing the most common causes of morbidity identified in p53P and Dp53P mice. (H) Representative H&E section of an acinar cell carcinoma (ACC) identified in a Dp53P mouse. Image taken at 10× magnification, scale bar: 100 µm. (I) Representative H&E section of an osteosarcoma identified in a p53P mouse. Image taken at 10× magnification, scale bar: 100 µm. (J) A single PanNET identified in a Dp53P mouse with representative H&E and immunohistochemical staining for synaptophysin. Images taken at 20× magnification, scale bars: 100 µm. (K) Average islet size per mouse presented relative to the average islet size of WT mice from Fig. 1D. (L) Representative H&E sections of pancreatic islets from mice of the different genotypes. Images taken at 10× magnification, scale bars: 100 µm.

Although we observed no evidence of oncogenic cooperation between *Men1* loss and loss of either *Daxx* or *Atrx* in mice, we wanted to further explore the potential for other mechanisms of genetic cooperation. We therefore chose to evaluate *Daxx* loss in cooperation with loss of two potent tumor suppressors: *Pten* and *p53* (Fig. 4A). PanNETs exhibit recurrent mutations and copy number losses in *PTEN* as well as *TSC1/2*, leading to activation of PI3K and mTOR signaling (Scarpa et al., 2017; Jiao et al., 2011). This signaling axis plays a functionally important role in tumor maintenance, which is likely underestimated by somatic mutation frequencies alone as everolimus (an mTOR inhibitor) significantly prolongs progression-free survival in patients with progressive advanced PanNETs compared to placebo-treated controls (Yao et al., 2011). Moreover, a recent publication demonstrated that homozygous *Pten* loss cooperated with *Men1* loss in β cells to accelerate pancreatic neuroendocrine tumorigenesis in mice (Wong et al., 2020). We established a cohort of *Pten^fl/fl^Pdx1-Cre^Tg^* (PP) and *Daxx^fl/fl^Pten^fl/fl^Pdx1-Cre^Tg^* (DPP) mice, with some having the *Rosa26^LSL-tdTomato^* reporter. Our choice of *Pdx1-Cre^Tg^* for these experiments was twofold. First, this line leads to Cre activation in pancreas progenitors and subsequently leads to gene deletion in all epithelial cells of the pancreas, including α cells as previously described. Second, *RIP-Cre* alleles have activity in the pituitary and can cause early morbidity, which is not present with *Pdx1-Cre^Tg^*. DPP mice have a median survival of 466 days, which is similar to that of PP mice (485 days) (Fig. 4B). The primary cause of morbidity in this cohort was associated with wasting and abdominal distension. Tissue samples from 11 DPP mice and eight PP mice were sent for histological evaluation. The pancreas revealed no evidence of pancreatic neuroendocrine tumorigenesis (Fig. 4C,D). We observed that 7/11 (64%) pancreases from DPP mice and 2/8 (25%) pancreases from PP mice presented with metaplasia, precancerous exocrine lesions and cystic lesions, as previously described in PP mice (Fig. 4C) (Stanger et al., 2005). Pronounced pathological changes were also observed in the liver. All 11 (100%) DPP mice had liver lesions, five of which were HCCs, and 7/8 (88%) PP mice had liver lesions, six of which were HCCs. These lesions were similar to the tumors seen in AMP mice and included tumors containing fat droplets and ballooning degeneration, mimicking steatohepatitis (Fig. 4E). This is similar to the observation of HCCs previously described in *Pten^fl/fl^Alb-Cre^Tg^* mice (Horie et al., 2004) and provides another example suggesting *Pdx1-Cre^Tg^* activity outside the pancreas; however, future studies are required to investigate cell-autonomous versus non-cell-autonomous contributions to the development of HCC in these models. We also identified gastric adenocarcinomas in one PP and two DPP mice, consistent with previous data indicating *Pdx1-Cre^Tg^* activity in the antral stomach (Magnuson and Osipovich, 2013). These phenotypes were consistent between PP and DPP mice, and therefore dependent only on *Pten* loss. Thus, *Daxx* loss does not promote pancreatic neuroendocrine tumorigenesis in the background of *Pten* deficiency in mice.

Although *TP53* mutations are rare in PanNETs, the p53 pathway can be attenuated through other mechanisms (Wasylishen and Lozano, 2016). There is evidence of overexpression of essential negative regulations of the pathway MDM2, MDM4 and WIP1 (also known as PPM1D) in PanNETs (Hu et al., 2010). Moreover, *RIP-Tag* mice rapidly develop PanNETs, and the primary cellular targets of the SV40 large T antigen are p53 and Rb (Hanahan, 1985). These data combined suggest that inhibition of the p53 pathway contributes to pancreatic neuroendocrine tumorigenesis in both humans and mice. We took a similar approach and established a cohort of *p53^fl/fl^Pdx1-Cre^Tg^* (p53P) and *Daxx^fl/fl^p53^fl/fl^Pdx1-Cre^Tg^* (Dp53P) mice; these mice had median survivals of 556 days and 581 days, respectively (Fig. 4F). Morbidity in both genotypes was most often associated with abdominal distension, wasting or palpable limb tumors and paralysis. On necropsy, abdominal and pancreatic tumors were isolated, and most were determined to be acinar cell carcinomas (ACCs) (Fig. 4G,H). These are highly cellular tumors containing cells that have large, relatively uniform nuclei with prominent nucleoli and granular, eosinophilic cytoplasm, exhibiting multiple architectural patterns, including acinar (mimicking normal pancreatic acini), solid nests/sheets and trabecular, and are frequently associated with tumor necrosis and numerous mitoses (Fig. 4H). These data indicate that *p53* loss alone is sufficient to drive ACCs *in vivo*, supporting previous reports (Rowley et al., 2011). The unexpected outcome of this cohort was the observation of frequent tumors on the limbs, which histopathological analysis revealed to be osteosarcomas (Fig. 4G,I). *p53* loss is strongly associated with osteosarcomagenesis (Pourebrahim et al., 2017; Overholtzer et al., 2003; Chen et al., 2014), and these data indicate previously unreported *Pdx1-Cre^Tg^* activity in an osteosarcoma precursor cell, revealed due to the robust consequences of homozygous *p53* loss. Other malignancies were also identified, including lymphomas in three Dp53P and one p53P mouse, and are included as other tumors (Fig. 4G). We further conducted comprehensive histopathological analysis of the pancreas and pancreas lesions, and we identified a single macroscopic PanNET lesion in this cohort (Fig. 4J) in one Dp53P mouse, which was hypoglycemic, with a blood glucose measurement of 15 mg/dl at necropsy. However, there were no significant changes in average islet size (Fig. 4K,L), further demonstrating that Daxx is not a robust tumor suppressor in the murine pancreas.

## DISCUSSION

Although DAXX and ATRX have both independent and shared functions (Mahmud and Liao, 2019; Valenzuela et al., 2021), the mutually exclusive pattern of mutations in PanNETs strongly implicates their shared function in tumor suppression. As a chaperone complex for histone 3.3, DAXX and ATRX contribute to the epigenetic regulation and silencing of heterochromatin, with noted roles at telomeres, centromeres and repeat loci (Elsasser et al., 2012; Elsasser et al., 2015; He et al., 2015; Lewis et al., 2010; Wasylishen et al., 2020; Sadic et al., 2015).

We believe that the lack of pancreatic neuroendocrine tumorigenesis observed across our cohorts is due to fundamental differences between mouse and human genomes. First, there are differences between human and inbred mouse telomeres. Data from human PanNETs indicate that *DAXX* or *ATRX* mutation strongly associates with activation of the ALT (Heaphy et al., 2011). This is a recombination-based and telomerase-independent mechanism of telomere maintenance, and this association implicates telomere dysfunction as a potential mechanism underlying tumorigenesis. Inbred mouse strains have long hypervariable telomere lengths, up to 150 kb (Kipling and Cooke, 1990). This is much longer than the telomere lengths of human cells, which average ∼10 kb. This difference has contributed to a lack of faithful modeling of other human diseases in mouse. The development of telomerase knockout mice (Blasco et al., 1997) allows genetic alterations to be evaluated in the context of shortened telomeres that more closely resemble human. For example, a mouse model of Duchenne muscular dystrophy lacking functional dystrophin (similar to patients) exhibits only a mild skeletal muscle phenotype and lacks a cardiac phenotype (Bulfield et al., 1984; Chapman et al., 1989). However, when crossed to telomerase-deficient mice for two generations, the dilated cardiomyopathy found in patients also becomes evident in the mice (Chang et al., 2016). A similar dependence of limiting telomere length was also observed in a mouse model of Werner syndrome (Chang et al., 2004). Telomere shortening, however, requires multiple generations of homozygous telomerase deficiency and can take years to achieve.

Another major difference between mouse and human genomes is the non-coding compartment, including transposable elements (TEs). TEs comprise ∼37% and 45% of mouse and human genomes, respectively, and have diverged over tens of millions of years of evolution. Endogenous retroviruses (ERVs) are one type of TE, and DAXX and ATRX have an established role in silencing these loci (Elsasser et al., 2015; He et al., 2015; Sadic et al., 2015). Our recent work demonstrated that this is an important function of Daxx *in vivo*, and that loss of ERV repression can lead to dysregulated expression of nearby protein-coding genes (Wasylishen et al., 2020). We further demonstrated that the most significant DAXX-dependent gene expression changes in human PanNETs were in genes closely located to a large human ERV locus. Although ERVs are abundant in both mouse and human genomes, the elements themselves and their locations in the genome are not conserved. So, although loss of ERV repression is likely a common feature of both genomes with *DAXX* or *ATRX* mutation, the downstream protein-coding genes affected will not be the same and neither will the subsequent effects on downstream cellular biology.

Thus, although many GEMMs reproduce tumor phenotypes that resemble those of human patients (*TP53* mutations are the perfect example), those tumor suppressors that impact human-specific features (telomere length, retroviral insertions) will not be easily recapitulated in mice.

## MATERIALS AND METHODS

### Mice

All mouse experiments were performed in compliance with National Institutes of Health (NIH) guidelines and Association for Assessment and Accreditation of Laboratory Animal (AAALAC) accreditation standards for animal research and approved by The University of Texas MD Anderson Cancer Center Institutional Animal Care and Use Committee. Mice were housed in individually ventilated cages, on individually ventilated cage racks, with up to five animals per cage after weaning. Direct cage bedding consisted of corn cob, Enviro-dri, Enrich-n-nest and nestlets. The facility uses a 14 h light (07:00-21:00) and 10 h dark (21:00-07:00) cycle. Mice were fed a standard diet sterilized by irradiation (PicoLab Rodent Diet 5053, Purina) and were provided reverse osmosis chlorinated or acidified water *ad libitum*.

*Daxx^fl^*, *Atrx^fl^*, *Men1^fl^* (The Jackson Laboratory, stock #005109), *p53^fl^*, *Pten^fl^*, *RIP-Cre^Tg^* (The Jackson Laboratory, stock #003573), *Rosa26^LSL-tdTomato^* (The Jackson Laboratory, stock #007914) and *Pdx1-Cre^Tg^* (The Jackson Laboratory, stock #014647) mice have been previously described (Wasylishen et al., 2020; Garrick et al., 2006; Zheng et al., 2008; Marino et al., 2000; Postic et al., 1999; Hingorani et al., 2003; Madisen et al., 2010; Crabtree et al., 2003). Genotyping primers are provided in Table S1. Mice were maintained on a mixed background and both Cre-positive and Cre-negative controls were used as indicated. Additionally, each independent cohort used its own littermate controls.

For the radiation cohort, 8- to 10-week-old mice were treated with one sublethal dose of 6 Gy in a Cesium-137 irradiator. For the pancreatitis cohort, 8- to 10-week-old mice were treated with six intraperitoneal injections of caerulein (1.25 μg) administered hourly on each of 2 consecutive days (12 total). The same treatment regime was repeated a total of three times with a 2-month interval between treatments. To confirm the induction of caerulein-induced pancreatitis (Fig. S3), mice were treated with six intraperitoneal injections of caerulein (1.25 μg) or PBS administered hourly on each of 2 consecutive days, and pancreases were collected 48 h after the final injection.

Across our cohorts, Kaplan–Meier plots represent overall survival with deaths (either by necropsy or spontaneous) plotted as events. Mice were right censored if they were alive at the 2-year end point of the cohort, and a small number were censored if they were prematurely removed from the study for welfare concerns unrelated to study end points. These included fight wounds, prolapses and buphthalmos. Additionally, a small number of mice from the caerulein-induced pancreatitis cohort were censored as they were sacrificed at early time points to evaluate inflammation.

### Histology, immunohistochemistry and immunofluorescence

Tissues harvested from mice were fixed in 10% neutral buffered formalin and embedded in paraffin. Four-micrometer sections were stained with Hematoxylin and Eosin (H&E) and examined using light microscopy. Tissue processing, paraffin embedding, sectioning, and H&E staining were performed by The University of Texas MD Anderson Cancer Center Department of Veterinary Medicine and Surgery Histology Laboratory. Immunohistochemistry was performed using standard methods, with Tris-EDTA buffer (pH 9.0) for antigen retrieval, and stained with an antibody against synaptophysin (ab32127, Abcam, 1:600). Visualization was performed using ABC and DAB kits (Vector Laboratories), and slides were counterstained with Hematoxylin. Histological sections were imaged on a Nikon Eclipse Ni microscope equipped with a color camera.

Immunofluorescence was performed using standard methods with citrate buffer (pH 6.0) for antigen retrieval. Primary rabbit antibody against red fluorescent protein (RFP) (600-401-379, Rockland, 1:500) and primary mouse antibody against glucagon with conjugated Alexa Fluor 488 (sc-514592 AF488, Santa Cruz Biotechnology, 1:200) were used in pancreas samples. Secondary anti-rabbit antibody, Alexa Fluor 555 (A-31572, Invitrogen, 1:1000) and 4′,6-diamidino-2-phenylindole (DAPI; 2 µg/ml) were used for visualization.

### Fluorescence microscopy

Confocal images of pancreas samples were acquired using a Nikon A1 confocal with hybrid detector and laser. The objective was Plan Apo VC 20× DIC N2 with a pixel size of 0.62 µm and a dwell time of 2.4 ms. We used three laser lines: 405 nm for DAPI, 488 nm for glucagon and 561 nm for RFP. Collection emission filters were 450/50 nm, 525/50 nm and 595/50 nm. *Z*-stack acquisition was taken at 0.88 µm. The pinhole was set at 1 Airy unit.

### Image quantification

Islet size was quantified from whole-pancreas sections scanned on an Aperio Scan Scope XT (Leica Biosystems) and manually annotated using Aperio ImageScope software (Leica Biosystems). The average islet sizes were calculated for each sample and normalized to values for Cre-negative control mice, with the data for WT mice presented in Fig. 1D. For this analysis, total endocrine area (both normal and tumor) of the pancreas was evaluated at end point. Macroscopic lesions identified in *Men1* mice at necropsy were dissected from the pancreas and not included in this analysis.

### Western blotting

Tissue samples were flash frozen, pulverized and lysed in sodium dodecyl sulfate (SDS) lysis buffer (1% SDS, 6.5 mM Tris-HCl pH 6.8, 25% glycerol, 10% β-mercaptoethanol). Protein extracts were separated by SDS/polyacrylamide gel electrophoresis (PAGE), transferred to nitrocellulose membranes, and probed with antibodies against Daxx (M-112, Santa Cruz Biotechnology, 1:1000), Atrx (H-300, Santa Cruz Biotechnology, 1:500), Men1 (A300-105A, Bethyl Laboratories, 1:1000), vinculin (V9131, Millipore Sigma, 1:1000) and actin (12004163, Bio-Rad, 1:5000). Proteins were visualized using Li-COR secondary antibodies and imaged using a ChemiDoc System (Bio-Rad). Signal was quantified using ImageJ software (NIH).

### Statistical analysis

Data are presented as means±s.d. All statistical analyses were performed using GraphPad Prism 9 software, and *P*<0.05 was considered statistically significant. Comparisons between two groups were made using unpaired, two-tailed Student's *t-*test, and comparisons among multiple groups were made using analysis of variance (ANOVA) with the Dunnett's multiple comparisons test to compare experimental samples to WT controls.

## Supplementary Material

Supplementary information
