# Supplementary material for: Context matters – Daxx and Atrx are not robust tumor suppressors in the murine endocrine pancreas
Source: Dis Model Mech. 2022 Aug 26;15(8):dmm049552. doi: 10.1242/dmm.049552 (PMC9438929; doi:10.1242/dmm.049552)
Supplement: Supplementary information [file dmm-15-049552-s1.pdf]

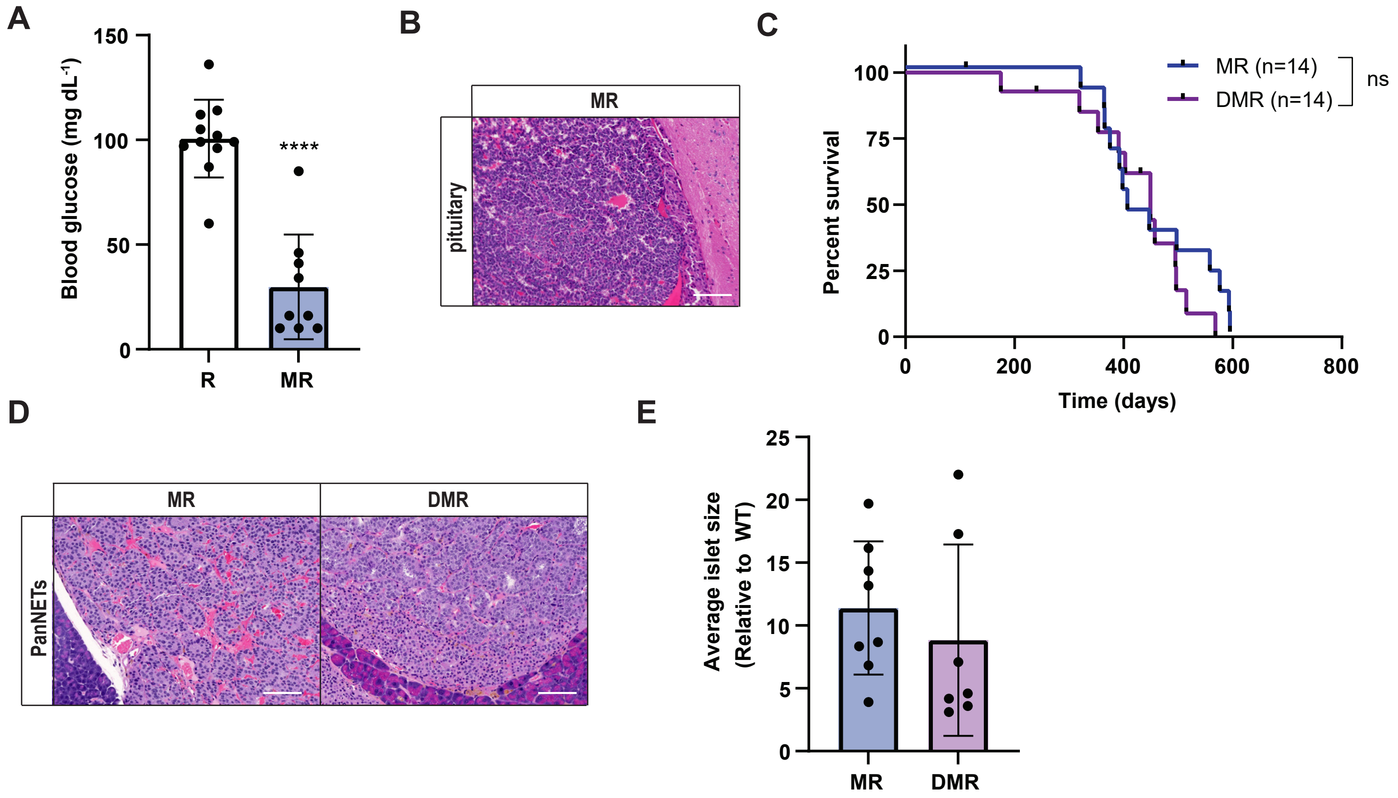

Fig. S1. Daxx loss from β-cells does not accelerate *Men1* loss-driven PanNETs

A) Blood glucose measurements at necropsy. R, *RIP-Cre*^{Tg}; MR, *Men1*$^{fl/fl}$*RIP-Cre*^{Tg}; ****P < 0.0001, Student's t-test. B) Representative hematoxylin and eosin (H&E) sections of a pituitary tumor from a MR mouse. Image taken at 10x magnification, bar = 100 µm. C) Kaplan-Meier survival analysis of MR (same data presented in Figure 1B) and *Daxx*$^{fl/fl}$*Men1*$^{fl/fl}$*RIP-Cre*^{Tg} (DMR) mice. ns, not significant, ****P<0.0001, Log-rank (Mantel-Cox) test. D) Representative H&E sections of pancreatic neuroendocrine tumors from MR and DMR mice. Images taken at 10x magnification, bar = 100 µm. E) Average islet size per mouse presented relative to the average size of wild-type mice from Figure 1D.

Disease Models & Mechanisms • Supplementary information

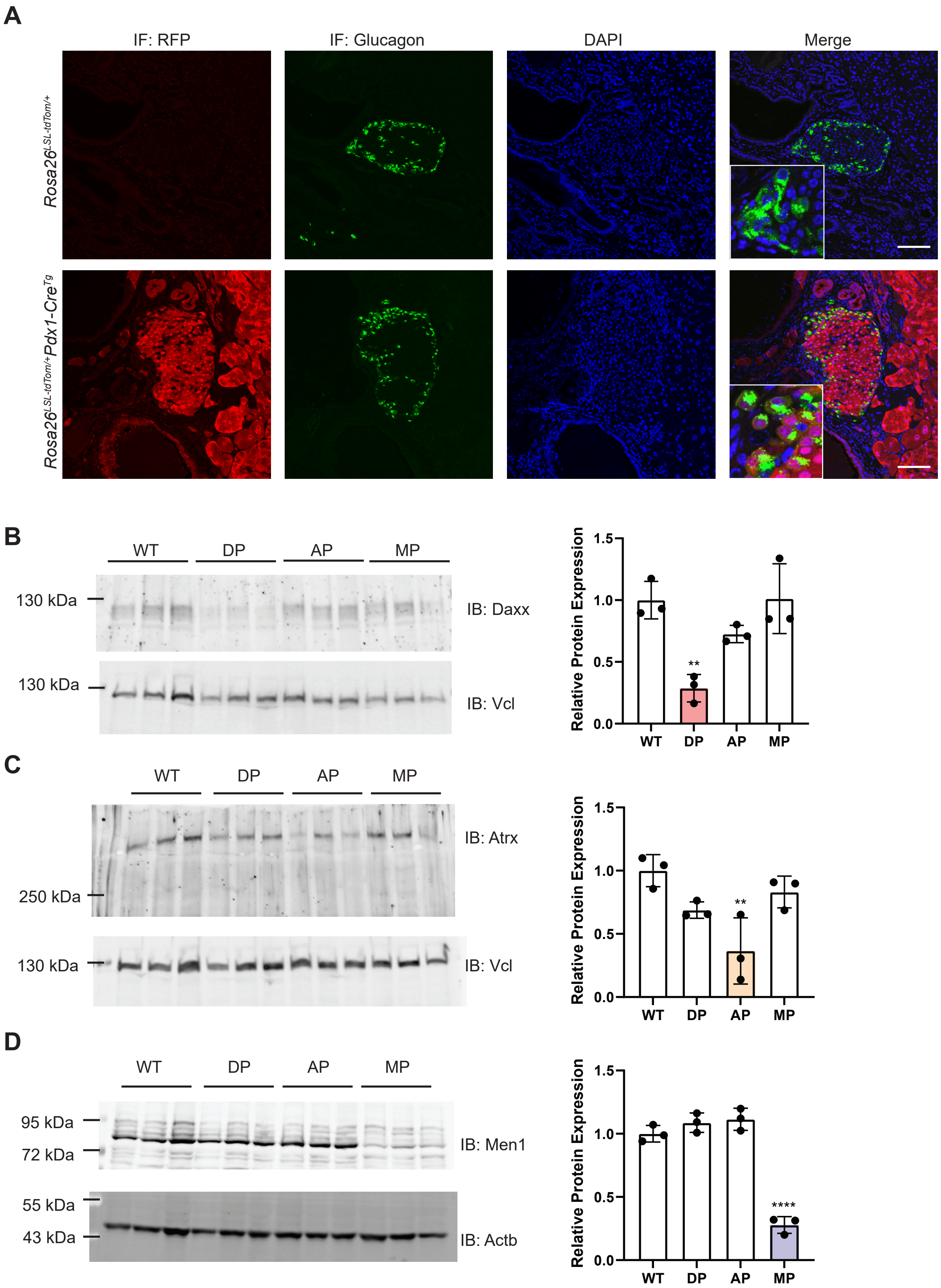

Fig. S2. Model validation confirms α cell targeting and efficient gene loss with *Pdx1-Cre*^{Tg}

A) Immunofluorescence analysis of co-expression of glucagon (α cell maker) and tdTomato in the pancreas of *Rosa26*$^{LSL\text{-}tdTom/+}$*Pdx1-Cre*^{Tg} mice. *Rosa26*$^{LSL\text{-}tdTom/+}$ pancreas is used as a negative control for tdTomato staining. Images taken at 20x magnification, bar = 100 µm. B-D) Western blot analysis of total pancreas lysates with quantifications. WT, wild-type; DP, *Daxx*$^{fl/fl}$*Pdx1-Cre*^{Tg}; AP, *Atrx*$^{fl/fl}$*Pdx1-Cre*^{Tg} or *Atrx*$^{fl/Y}$*Pdx1-Cre*^{Tg}; MP, *Men1*$^{fl/fl}$*Pdx1-Cre*^{Tg}. **P<0.01, ****P<0.0001, one-way ANOVA with Dunnett's multiple comparisons test compared with WT. Vcl, vinculin; Actb, β-actin.

Disease Models & Mechanisms • Supplementary information

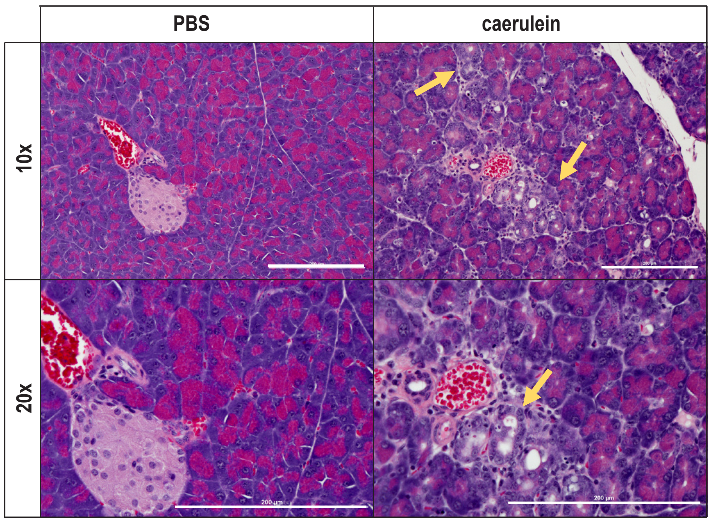

Fig. S3. Pancreatitis is induced after caerulein treatment

Representative hematoxylin and eosin (H&E) sections of pancreas from *Daxx*$^{fl/fl}$*RIP-Cre*^{Tg} (DR) 48 hours after caerulein treatment to induce pancreatitis or PBS control. Images taken at 10x and 20x magnification, bar = 200 µm. Examples of acinar to ductal metaplasia are indicated with yellow arrows.

Table S1. Primer sequences used in this study

Purpose	Allele/Gene	Primer 1 (5'-3')	Primer 2 (5'-3')	Primer 3 (5'-3')
Genotyping	*Atrx*^{fl}	AGCATGACAAGGTGTATGTGGA	ACCCAGTCCAAGAAATGAAGCA	
Genotyping	*Daxx*^{fl}	AGCAGTAACTCCGGTAGTAGGAAG	AGGAACGGAACCACCTCAG	
Genotyping	*Men1*^{fl}	CCCACATCCAGTCCCTCTTCAGCT	CCCTCTGGCTATTCAATGGCAGGG	CGGAGAAAGAGGTAATGAAATGGC
Genotyping	Generic Cre	TCCAATTTACTGACCGTACACCAA	CCTGATCCTGGCAATTTCGGCTA	
Genotyping	*Internal control*	CTAGGCCACAGAATTGAAAGATCT	GTAGGTGGAAATTCTAGCATCATCC	
Genotyping	*Pdx1-Cre*^{Tg}	CCTGGACTACATCTTGAGTTGC	AGGCAAATTTTGGTGTACGG	
Genotyping	*Pten*^{fl}	GGAGCATGTCTGGCAATG	GGAAGAGGGTGGGGATAC	
Genotyping	*p53*^{fl}	AAGGGGTATGAGGGACAAGG	GAAGACAGAAAAGGGGAGGG	
Genotyping	*Rosa26*$^{LSL\text{-}tdtomato}$	GGCATTAAAGCAGCGTATCC	CTGTTCCTGTACGGCATGG	
		AAGGGAGCTGCAGTGGAGTA	CCGAAAATCTGTGGGAAGTC	
